# Pulmonary Embolism as the Initial Presentation of Klippel-Trénaunay Syndrome

**DOI:** 10.1016/j.jaccas.2026.109228

**Published:** 2026-07-13

**Authors:** Ahmed Al Ghazawy, Shriya Bavishi, Akhilesh Jain, Joseph Ingrassia

**Affiliations:** Department of Cardiology, Hartford Hospital, Hartford HealthCare, Hartford, Connecticut, USA

**Keywords:** catheter-directed thrombolysis, congenital vascular malformation, deep venous thrombosis, Klippel-Trénaunay syndrome, *PIK3CA*-related overgrowth spectrum, pulmonary embolism

## Abstract

**Background:**

Congenital vascular malformations are an under-recognized cause of venous thromboembolism in young adults, and their early identification carries important management implications.

**Case Summary:**

A 23-year-old man presented with intermediate-high risk pulmonary embolism (PE) and right ventricular dysfunction in the context of lifelong unilateral lower extremity varicosities. After classification by the PE response team, catheter-directed thrombolysis with EKOS ultrasound-assisted catheters achieved hemodynamic stabilization. Subsequent magnetic resonance venography identified extensive venous malformations, embryonic lateral vein persistence, and osseous involvement of the femoral medullary cavity, establishing a diagnosis of Klippel-Trénaunay syndrome (KTS) within the *PIK3CA*-related overgrowth spectrum. The patient was transitioned to long-term direct oral anticoagulation and referred for genetic counseling and *PIK3CA* mutation testing.

**Discussion:**

KTS, a *PIK3CA*-related overgrowth spectrum disorder caused by somatic *PIK3CA* gain-of-function mutations, confers elevated lifelong venous thromboembolism (VTE) risk because of dysmorphic venous architecture and abnormal perforator veins. Recognition of this syndrome in young patients with VTE is essential, as it directs structured long-term surveillance, individualized thromboprophylaxis, and eligibility for emerging PI3K-AKT-mTOR targeted therapies.

**Take-Home Messages:**

Young patients with unprovoked PE and unilateral, early onset varicosities warrant evaluation for congenital vascular malformations. Identifying KTS enables multidisciplinary management and long-term VTE risk reduction.

## Visual Summary

**Figure:**
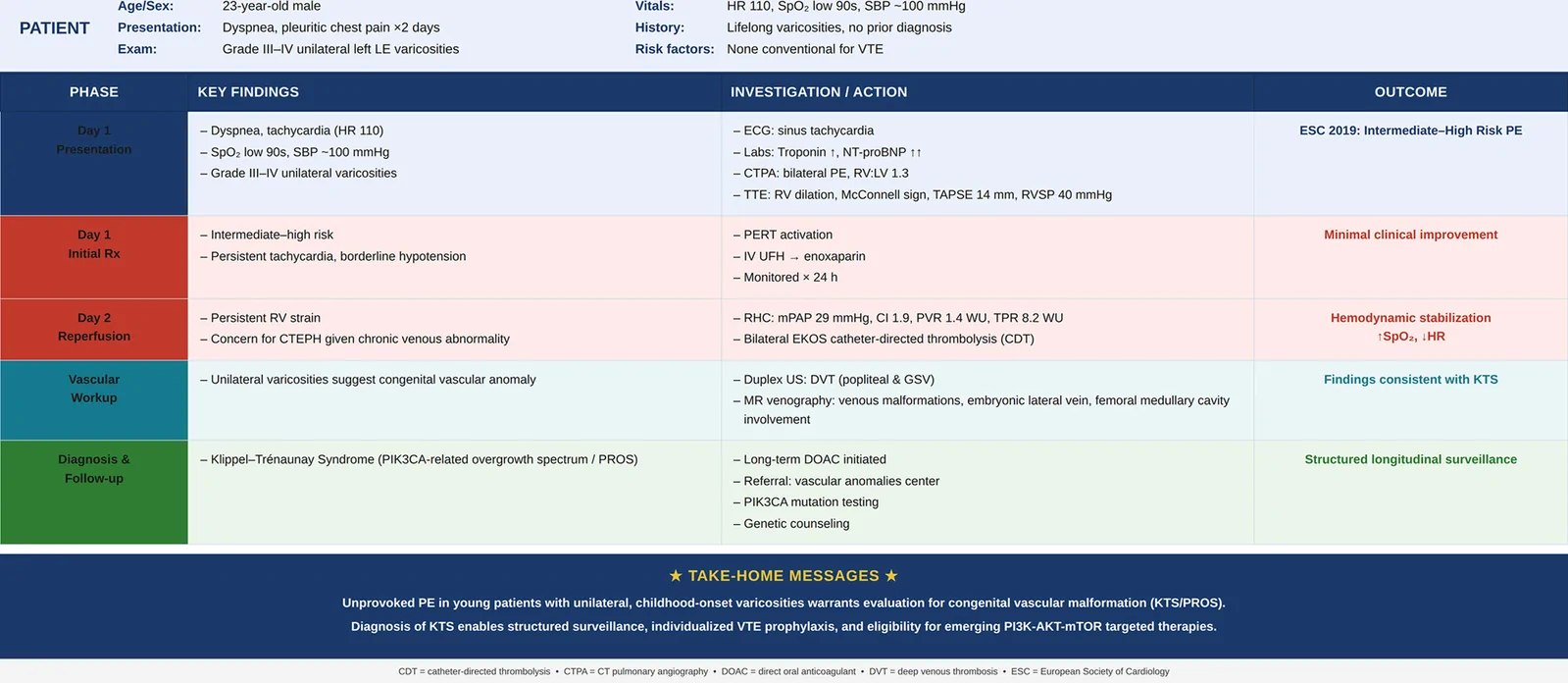

**Visual Summary:**
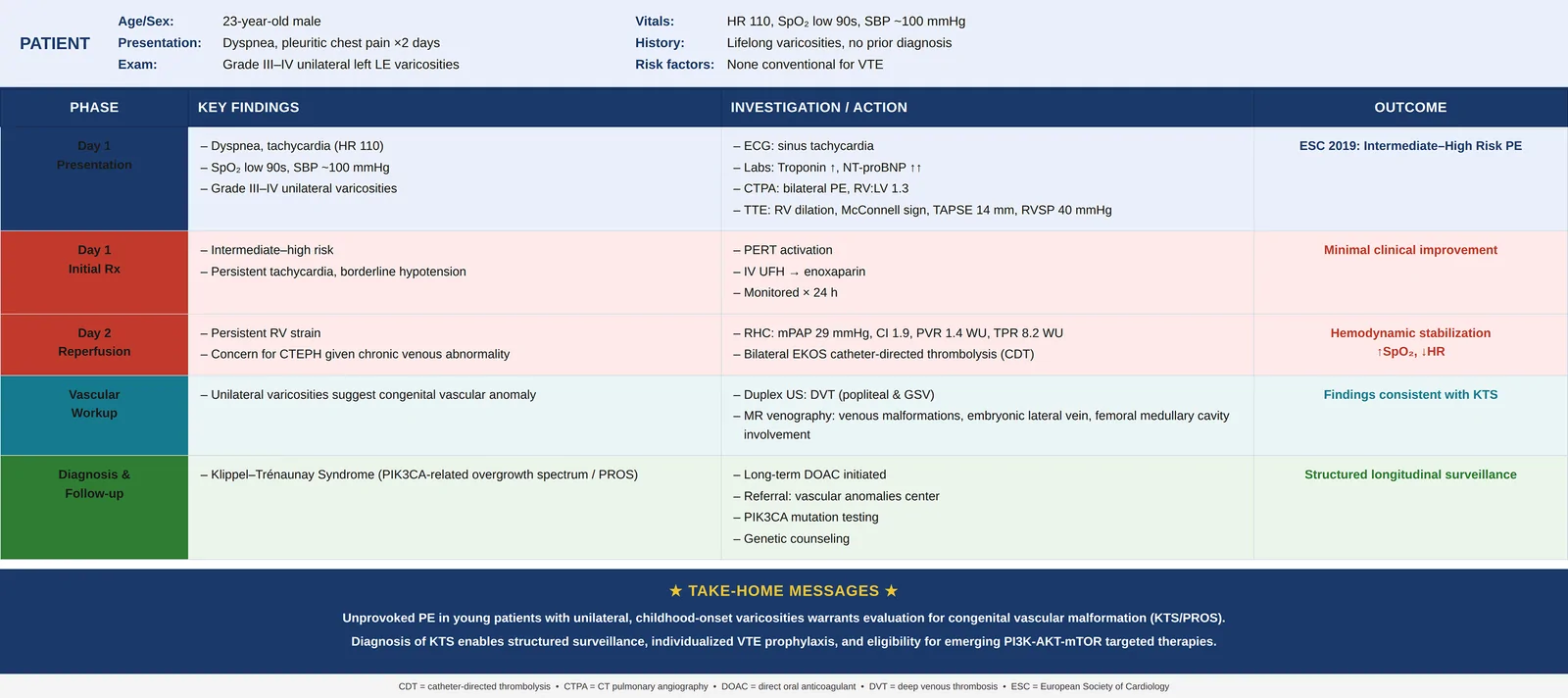
Clinical Presentation, Diagnostic Evaluation, and Management Timeline of Klippel-Trénaunay Syndrome as an Underlying Cause of Intermediate- to High-Risk Pulmonary Embolism in a Young Patient The visual summary presents the patient's clinical course as a sequential timeline. The top section summarizes the patient's demographics, presentation, vital signs, examination findings, and risk factors. Each subsequent row corresponds to a phase of care—initial presentation with 2019 ESC risk stratification, initial anticoagulation, catheter-directed reperfusion, vascular workup, and final diagnosis with follow-up—paired with the key findings, investigations and actions, and outcomes of that phase. The bottom section highlights the take-home messages regarding evaluation for congenital vascular malformations in young patients with unprovoked pulmonary embolism and the management implications of a KTS/PROS diagnosis. CDT = catheter-directed thrombolysis; DOAC = direct oral anticoagulant; DVT = deep venous thrombosis; ESC = European Society of Cardiology; GSV = great saphenous vein; KTS = Klippel-Trénaunay syndrome; PROS = *PIK3CA*-related overgrowth spectrum; VTE = venous thromboembolism.

Venous thromboembolism (VTE) in young adults is uncommon, and its occurrence should prompt thorough evaluation for underlying predisposing conditions. Although inherited thrombophilias and acquired risk factors are well-recognized etiologies, congenital vascular malformations represent an underappreciated substrate for recurrent or atypically severe VTE. Klippel-Trénaunay syndrome (KTS) is a rare congenital disorder characterized by capillary malformations, venous and lymphatic anomalies, and soft tissue or bony hypertrophy of the affected limb. Patients with KTS harbor an elevated lifelong risk for deep venous thrombosis (DVT) and pulmonary embolism (PE) because of inherently dysmorphic venous architecture and impaired venous return.

We describe a case in which acute PE prompted the eventual diagnosis of KTS in a young adult with previously uncharacterized extensive varicosities, illustrating the diagnostic and therapeutic challenges this rare syndrome presents.

## History of Presentation

A 23-year-old man with no prior diagnosis of a clotting disorder presented to the emergency department with a 2-day history of progressive dyspnea, dizziness, and left-sided pleuritic chest pain worsening with inspiration. He denied lower extremity swelling, recent travel, prolonged immobilization, or personal or family history of thrombophilia. He worked a job requiring prolonged standing and reported occasional cannabis use, denying tobacco, alcohol, or intravenous drug use.

**Figure 1:**
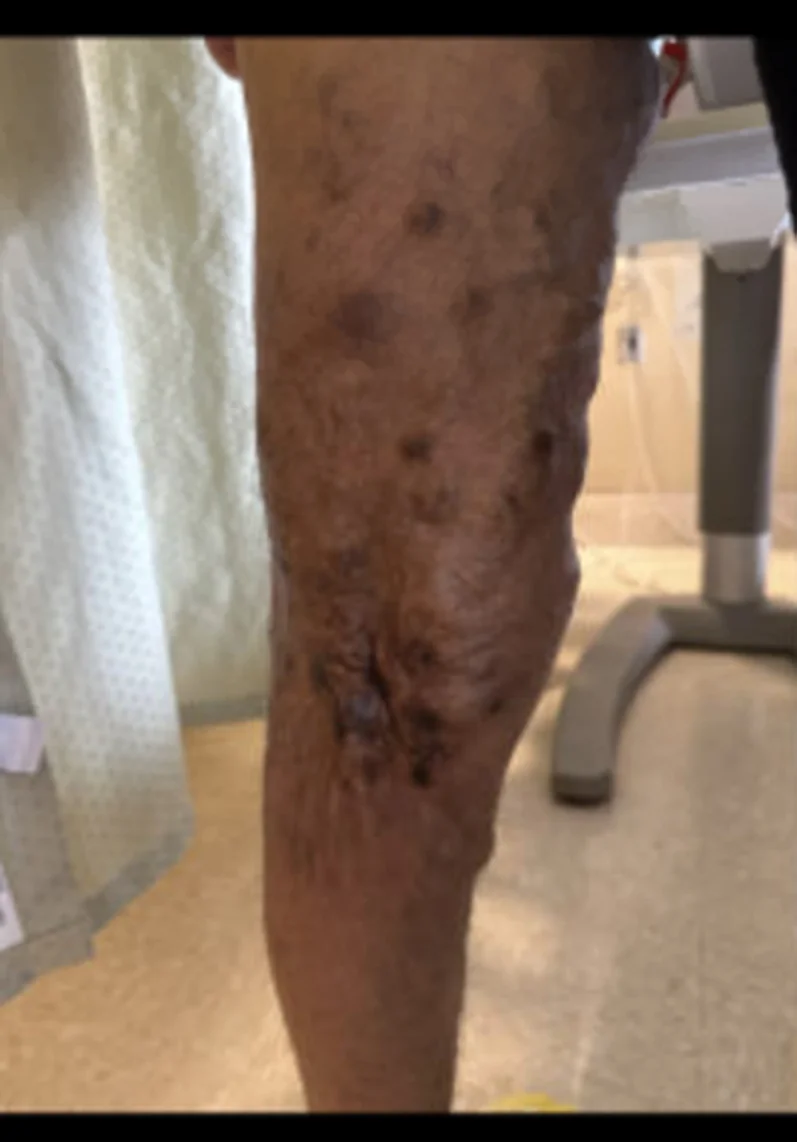
Clinical Photograph of the Left Lower Extremity Demonstrating Grade III-IV Varicosities With Nodular Morphology, Extending From the Calf to the Pelvic and Inguinal Region

On presentation, the patient was tachypneic and dyspneic with oxygen saturation in the low 90s on room air. He was tachycardic with a heart rate of 110 beats/min and had borderline systolic blood pressures in the low 100s mm Hg. Physical examination of the left lower extremity revealed grade III-IV varicosities extending from the calf to the pelvic and inguinal region, with a predominantly nodular morphology most prominent over the medial thigh and popliteal fossa. A firm, tender nodule was palpable in the popliteal region. A left scrotal varicocele was also noted. The right lower extremity was unremarkable. Clinical photographs are presented in Figure 1.

## Past Medical History

Pertinent medical history included extensive varicose veins confined to the left lower extremity, extending to the inguinal and scrotal region, reportedly present since birth and progressively worsening since adolescence. He had been evaluated previously without a definitive diagnosis and had been prescribed aspirin, which he was not taking consistently.

## Differential Diagnosis

The differential diagnosis for early onset, unilateral, extensive varicosities with thromboembolic complications in a young patient included 1) KTS/*PIK3CA*-related overgrowth spectrum (PROS) spectrum disorder, given the unilateral distribution, childhood onset, and extensive venous malformations; 2) May-Thurner syndrome (iliac vein compression), considered but deemed unlikely given childhood onset, absence of typical demographics, and lack of hemodynamically significant left common iliac vein obstruction on magnetic resonance (MR) venography; 3) Parkes-Weber syndrome, distinguished by the presence of arteriovenous fistulas and high-flow shunting—not seen here; and 4) nutcracker syndrome, not supported by the clinical picture and excluded. Inherited thrombophilia was also considered given unprovoked PE in a young patient; evaluation was planned after the acute hospitalization.

## Investigations

Electrocardiography demonstrated sinus tachycardia without significant ST or T-wave changes. Chest radiography was unremarkable. Laboratory evaluation revealed an elevated troponin level, normal lactate, and markedly elevated N-terminal pro–B-type natriuretic peptide.

**Figure 2:**
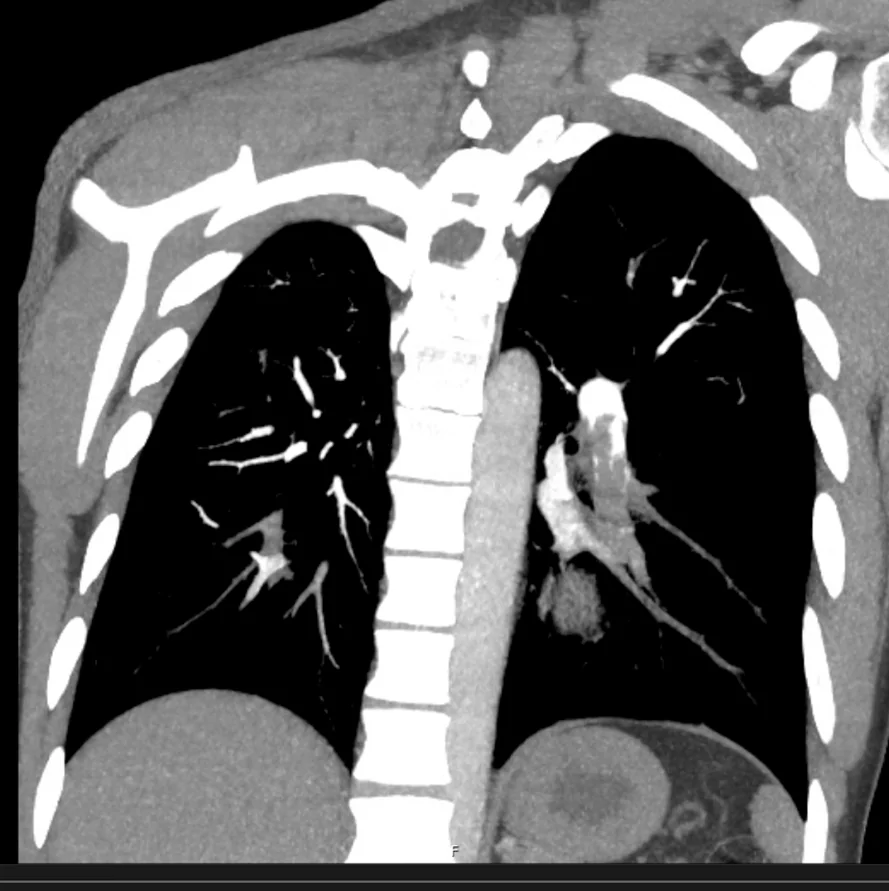
Computed Tomography Pulmonary Angiography Demonstrating Acute Pulmonary Embolism Involving the Main and Distal Pulmonary Arteries Bilaterally

Computed tomography pulmonary angiography (Figure 2) demonstrated acute PE involving the main and distal pulmonary arteries bilaterally, with a right ventricular–to–left ventricular (RV:LV) diameter ratio of 1.3, an enlarged right atrium and RV, and septal flattening—constituting the imaging signature of RV strain. Transthoracic echocardiography subsequently confirmed these findings, demonstrating normal LV size and systolic function, a dilated RV with an estimated tricuspid annular plane systolic excursion (TAPSE) of 14 mm, a positive McConnell sign, septal bowing, and moderate tricuspid regurgitation with an RV systolic pressure of 40 mm Hg.

The term “RV strain pattern” refers to a constellation of imaging and physiologic findings that reflect acute RV pressure overload from pulmonary vascular obstruction that was evident in this case, including RV dilation (RV:LV ≥1.0), septal bowing toward the LV, hypokinesis of the RV free wall with preserved apical contractility (McConnell sign on echocardiography), reduced systolic excursion of the tricuspid annulus, and biomarker evidence of myocardial stress (elevated troponin and natriuretic peptides). Its identification is prognostically important because it stratifies normotensive patients into a higher-risk category for short-term hemodynamic deterioration.

On the basis of this composite assessment, the patient was classified as having intermediate- to high-risk PE according to the 2019 European Society of Cardiology guidelines.^1^ The combination of RV dysfunction on imaging and a positive cardiac biomarker corresponds to the intermediate- to high-risk category in the European Society of Cardiology framework.

## Management

The PE response team was activated, and therapeutic anticoagulation with intravenous unfractionated heparin transitioned to enoxaparin was initiated promptly. Despite therapeutic anticoagulation, the patient demonstrated minimal clinical improvement: He remained tachycardic at a heart rate of 110 beats/min, persistently tachypneic and dyspneic at rest and with mild exertion, and continued to have borderline systolic blood pressures. Given his young age, the extensive clot burden on imaging, and concerns regarding the potential for progressive RV failure and the development of chronic thromboembolic pulmonary hypertension, particularly in the context of his long-standing congenital venous abnormality and the possibility of a superimposed chronic component to his thromboembolic disease, the multidisciplinary PE response team recommended advancing to reperfusion therapy. The decision for percutaneous reperfusion was made the day after his presentation.

Right heart catheterization was performed to define the hemodynamic profile and guide reperfusion strategy in the setting of suspected acute-on-chronic pulmonary vascular disease. Hemodynamics demonstrated elevated pulmonary arterial pressures (45/18 mm Hg; mean: 29 mm Hg), elevated pulmonary capillary wedge pressure (24 mm Hg), reduced cardiac index (1.9 L/min/m^2^), low pulmonary vascular resistance (1.4 Wood units), and elevated total pulmonary resistance (8.2 Wood units).

The relatively preserved pulmonary vascular resistance despite elevated mean pulmonary arterial pressure and total pulmonary resistance suggested acute mechanical pulmonary vascular obstruction rather than chronic fixed pulmonary vascular remodeling as seen in advanced chronic thromboembolic pulmonary hypertension. The low cardiac index with borderline-preserved blood pressure was consistent with normotensive cardiogenic shock due to acute RV failure.

The elevated pulmonary capillary wedge pressure was interpreted in the context of acute RV pressure overload rather than primary left-sided heart failure. Acute RV dilation likely caused interventricular septal shift with impaired LV filling (ventricular interdependence), while increased intrapericardial pressure from RV enlargement elevated measured LV filling pressures without true LV volume overload. Overall, the findings supported predominantly acute pulmonary vascular obstruction.

Although right heart catheterization in acute PE can be risky given limited RV reserve, it was safely performed here given preserved blood pressure, young age, immediate PE response team availability, and continuous monitoring.

**Figure 3:**
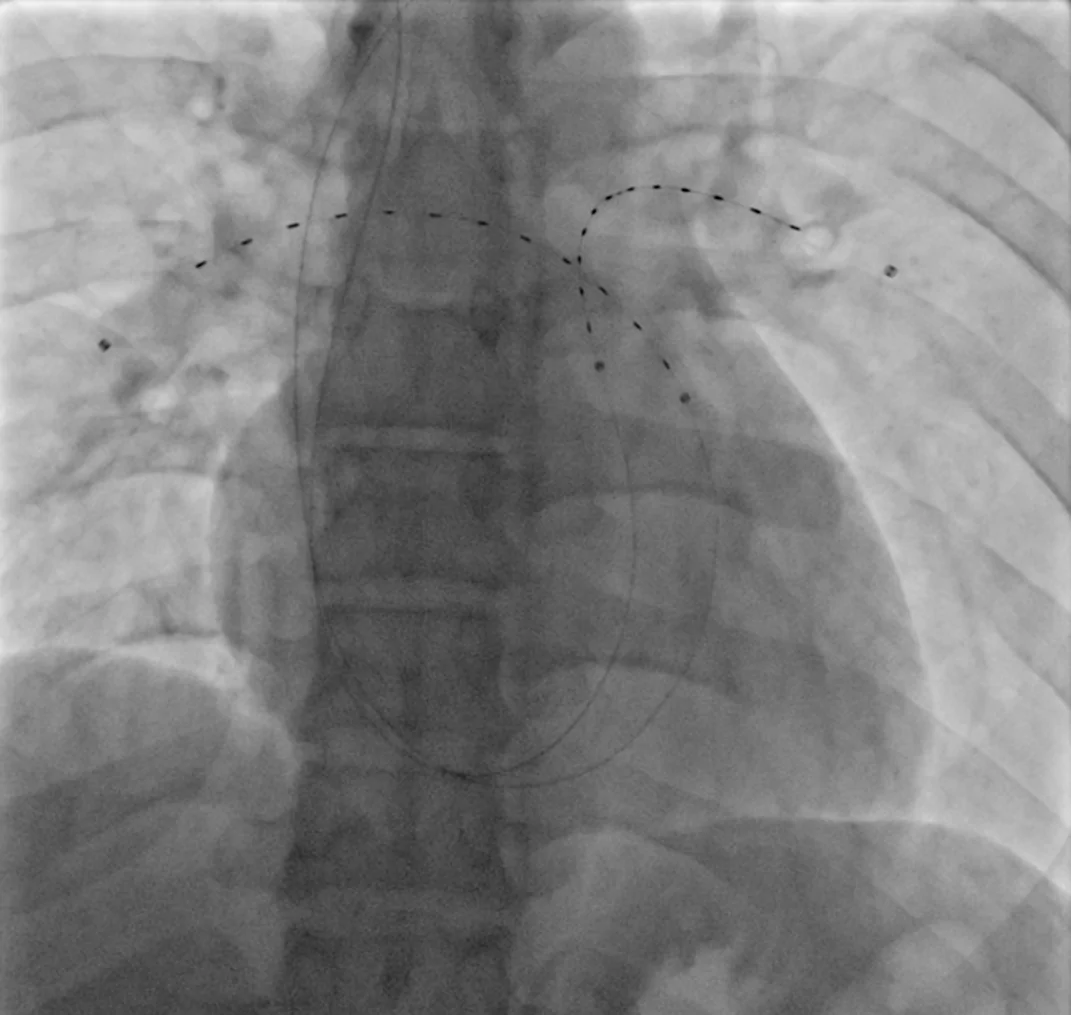
Fluoroscopic Images of Catheter-Directed Thrombolysis Using the EKOS Ultrasound-Assisted Catheter in Both Pulmonary Arteries

Catheter-directed thrombolysis (CDT) with bilateral EKOS ultrasound-assisted catheters (Figure 3) was chosen over mechanical thrombectomy because the patient had both proximal and distal segmental thrombus burden, making prolonged low-dose thrombolytic delivery more suitable for distal clot resolution. The patient had no contraindications to thrombolysis, and CDT allowed targeted thrombolytic delivery with reduced systemic exposure. Respiratory effort, oxygenation, and heart rate improved after treatment.

**Figure 4:**
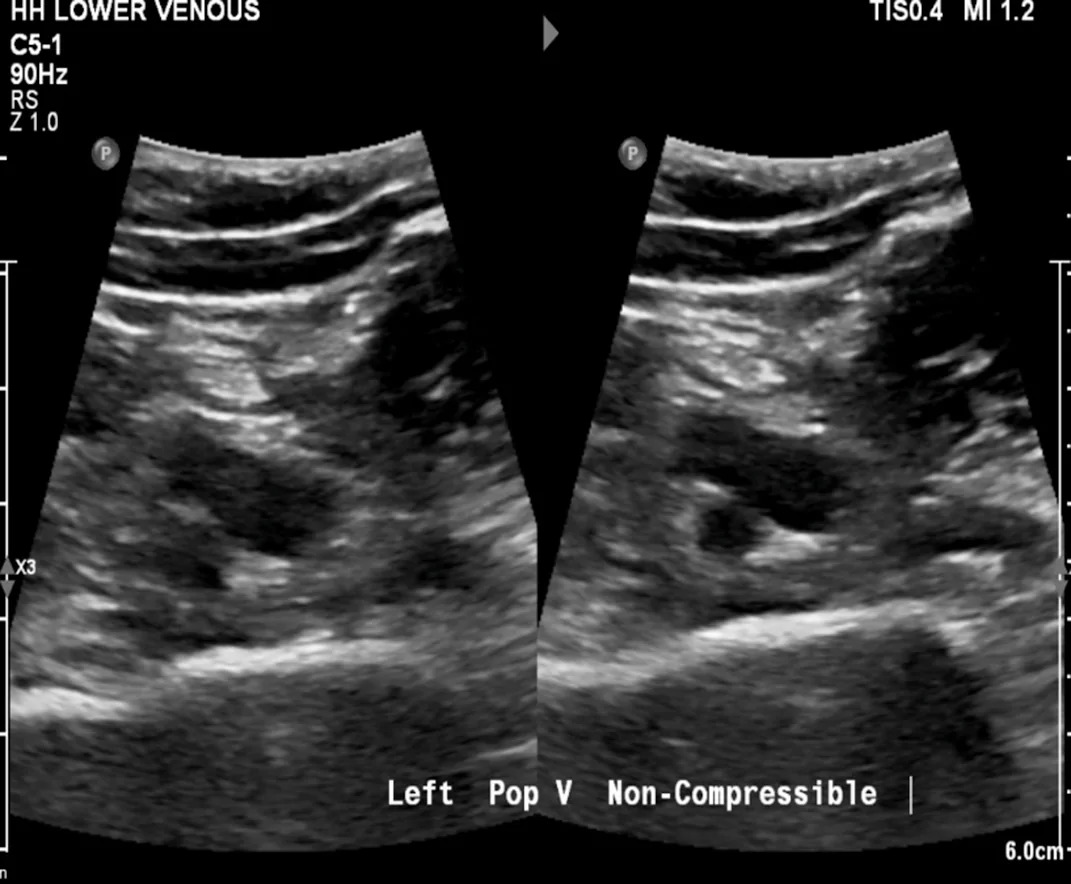
Venous Duplex Ultrasonography Demonstrating Acute Noncompressible Deep Vein Thrombosis Within the Popliteal Vein

Given the unilateral distribution and severity of varicosities, venous duplex ultrasonography was obtained and demonstrated acute DVT involving the popliteal vein and great saphenous vein from the mid-calf to the medial knee (Figure 4). Both superficial and deep venous systems were well developed.

**Figure 5:**
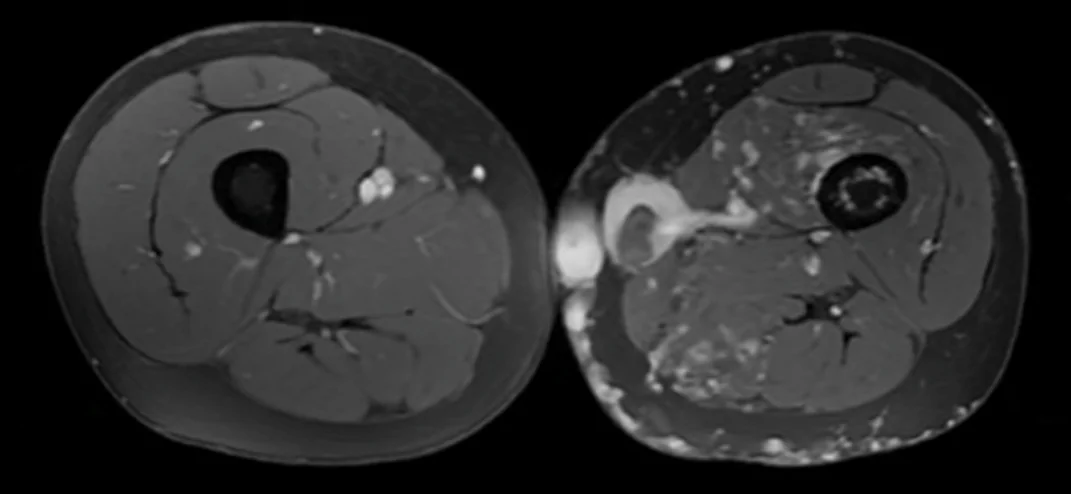
MR Venography of the Left Lower Extremity Demonstrating Mixed Macrocystic and Microcystic Venous Malformations, Great Saphenous Vein Ectasia, and an Abnormal Perforator Vein—Findings Consistent With KTS KTS = Klippel-Trénaunay syndrome; MR = magnetic resonance.

**Figure 6:**
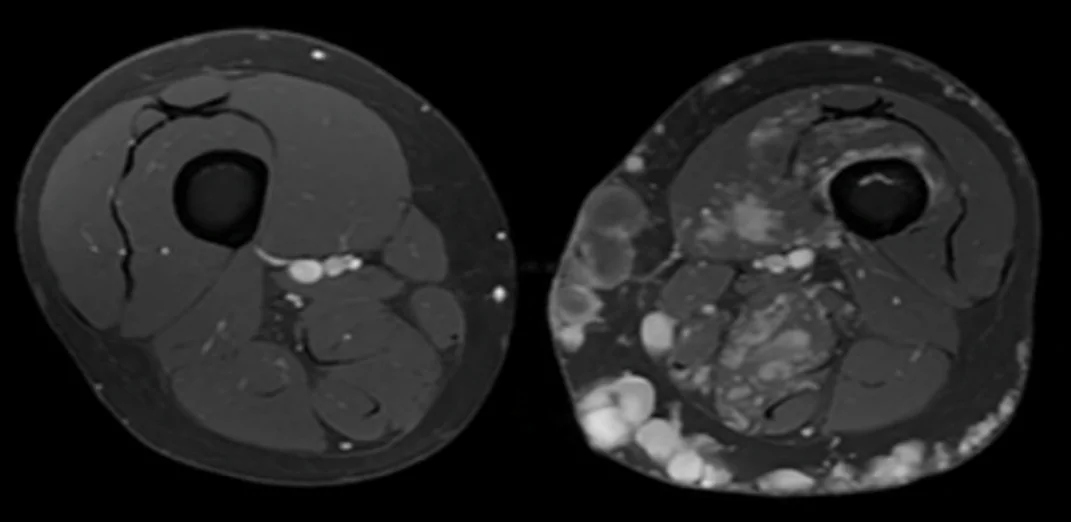
MR Venography of the Left Lower Extremity Demonstrating Mixed Macrocystic and Microcystic Venous Malformations Both Superficially and Intramuscularly, a Feature Suggestive of KTS KTS = Klippel-Trénaunay syndrome; MR = magnetic resonance.

MR venography of the pelvis and left lower extremity (Figures 5 and 6) demonstrated the following: a patent, normal-caliber inferior vena cava; left common iliac vein compression beneath the right common iliac artery of uncertain hemodynamic significance; great saphenous vein ectasia measuring up to 3.1 × 3.4 cm at the mid-femur with superficial venous thrombosis; an abnormal perforator vein connecting the distal femoral vein to the partially thrombosed saphenous vein; and extensive mixed macrocystic and microcystic venous malformations throughout the subcutaneous and intramuscular compartments of the left lower extremity, extending into the perineum and scrotum, with involvement of the femoral medullary cavity. These findings were collectively interpreted as consistent with the angio-osteohypertrophy family of syndromes, including KTS.

## Outcomes and Follow-Up

The patient demonstrated significant clinical improvement after CDT, with resolution of pulmonary hypertension on follow-up echocardiography. He was transitioned to long-term direct oral anticoagulation therapy. Given the high suspicion for an underlying PROS/KTS diagnosis, the patient was referred to a specialized vascular anomalies center for genetic counseling, *PIK3CA* mutation testing from affected tissue, and evaluation for ongoing multidisciplinary surveillance.

## Discussion

Primary varicose veins confer a measurable excess risk for VTE, with population-based data estimating an incidence of approximately 0.48 per 1,000 person-years for both DVT and PE—corresponding to a roughly 5-fold increased risk for DVT and 1.7-fold increased risk for PE compared to matched controls.^2^ However, the severity, unilateral distribution, and childhood onset of varicosities in this patient were incongruent with primary varicose vein disease, which typically manifests bilaterally in adulthood. The fact that this patient had previously been evaluated without a definitive diagnosis underscores a broader clinical point: Early recognition and timely referral of young patients with extensive or unilateral varicosities to a specialist familiar with congenital vascular malformations is essential, as missed or delayed diagnosis directly translates into preventable thromboembolic morbidity.

The differential diagnosis for early onset, unilateral, extensive varicosities should prioritize congenital vascular malformations. Congenital venous malformations are the most common vascular malformation, representing low-flow lesions composed of ectatic, dysmorphic venous channels that are present at birth but may not manifest clinically until childhood or adolescence.^3^ The angio-osteohypertrophy family of syndromes—classified within PROS—comprises congenital disorders caused by somatic mosaic gain-of-function mutations in the PI3K-AKT-mTOR signaling pathway, which regulates embryonic cellular proliferation and vascular development. KTS is the prototypical syndrome and classically presents with the triad of capillary malformation (port-wine stain), limb overgrowth, and venous varicosities, most commonly affecting a single lower extremity.^4^

Related syndromes include CLOVES syndrome and Parkes-Weber syndrome, the latter distinguished by the presence of arteriovenous fistulas and high-flow shunting. May-Thurner syndrome was considered but was deemed unlikely given childhood onset, absence of typical demographics, and MR venographic findings that did not demonstrate hemodynamically significant left common iliac vein obstruction.^5^ Nutcracker syndrome, presenting with hematuria, left flank pain, orthostatic proteinuria, and left-sided varicocele, was not supported by the clinical picture and was excluded.^6^

The constellation of unilateral varicosities with hemihypertrophy, mixed macro- and microcystic venous malformations involving subcutaneous and intramuscular compartments on magnetic resonance imaging, extension into the perineum and scrotum, and involvement of the femoral medullary cavity is pathognomonic for KTS within the PROS spectrum. Slow-flow characteristics—cystic venous channels without flow voids, absence of arterial feeders, lack of arterialized Doppler waveforms—distinguish this entity from Parkes-Weber syndrome. The persistence of the embryonic lateral vein, identified in up to 56% of KTS patients, was also present in this case and represents a pathognomonic feature.

VTE risk in KTS is multifactorial, arising from dysmorphic venous channels, venous stasis, and abnormal perforator veins connecting the superficial and deep systems. In this patient, the perforator vein connecting the distal femoral vein to the thrombosed great saphenous vein served as an independent DVT risk factor and a direct conduit for thrombus propagation to the pulmonary vasculature.

Acute management followed established PE guidelines, with PE response team activation, anticoagulation, and catheter-directed thrombolysis via EKOS catheters achieving hemodynamic stabilization. Long-term direct oral anticoagulation therapy was initiated given the high recurrence risk inherent to the underlying vascular malformation.

Importantly, the diagnostic value of recognizing an underlying KTS/PROS in a young patient with VTE extends beyond the acute hospitalization. It creates an opportunity for structured longitudinal follow-up, periodic reassessment of vascular anatomy, and individualized venous thromboembolism prophylaxis around recognized high-risk periods such as prolonged immobilization, surgery, and pregnancy—interventions that may meaningfully reduce the incidence of recurrent PE in this population. Confirmation of the underlying diagnosis is recommended via *PIK3CA* mutation testing from affected tissue using high-depth next-generation sequencing, with reported detection rates of 85% to 90%.^7^ Emerging targeted molecular therapies, including PI3K inhibitors (alpelisib) and mTOR inhibitors (sirolimus), have shown promising early efficacy in select patients with PROS, although robust randomized data are not yet available; their potential availability should not, however, be presented as an established standard of care.

## Conclusions

PE in young patients with extensive, early onset, unilateral varicosities should prompt systematic evaluation for an underlying congenital vascular malformation. Red flags warranting further work-up include childhood or adolescent onset, unilateral disease, extensive anatomical involvement, absence of conventional VTE risk factors, and thromboembolic complications disproportionate to patient age. MR venography and multidisciplinary evaluation involving hematology, vascular surgery, and a vascular anomalies specialist are essential. Early diagnosis enables structured surveillance and targeted VTE prophylaxis, and informs eligibility for emerging molecular therapies in the PI3K-AKT-mTOR pathway.

## Funding Support and Author Disclosures

The authors have reported that they have no relationships relevant to the contents of this paper to disclose.

**Take-Home Messages**

• Unprovoked or atypically severe pulmonary embolism in young patients should prompt evaluation for underlying congenital vascular malformations, particularly when accompanied by unilateral, early onset varicosities.
• Identifying Klippel-Trénaunay syndrome has important longitudinal management implications, including long-term anticoagulation, multimodality imaging surveillance, and tailored VTE prophylaxis to reduce recurrence risk.
